# The progress of multimodal imaging combination and subregion based radiomics research of cancers

**DOI:** 10.7150/ijbs.71046

**Published:** 2022-05-09

**Authors:** Luyuan Zhang, Yumin Wang, Zhouying Peng, Yuxiang Weng, Zebin Fang, Feng Xiao, Chao Zhang, Zuoxu Fan, Kaiyuan Huang, Yu Zhu, Weihong Jiang, Jian Shen, Renya Zhan

**Affiliations:** 1 Department of Neurosurgery, First Affiliated Hospital, School of Medicine, Zhejiang University, Hangzhou, Zhejiang, China.; 2 Department of Otolaryngology Head and Neck Surgery, Xiangya Hospital, Central South University, Changsha, Hunan, China.; 3 National Clinical Research Center for Geriatric Disorders, Xiangya Hospital, Changsha, Hunan, China.

**Keywords:** cancer, multimodal imaging, subregion, heterogenous, radiomics

## Abstract

In recent years, with the standardization of radiomics methods; development of tools; and popularization of the concept, radiomics has been widely used in all aspects of tumor diagnosis; treatment; and prognosis. As the study of radiomics in cancer has become more advanced, the currently used methods have revealed their shortcomings. The performance of cancer radiomics based on single-modality medical images, which based on their imaging principles, only partially reflects tumor information, has been necessarily compromised. Using the whole tumor as a region of interest to extract radiomic features inevitably leads to the loss of intra-tumoral heterogeneity of, which also affects the performance of radiomics. Radiomics of multimodal images extracts various aspects of information from images of each modality and then integrates them together for model construction; thus, avoiding missing information. Subregional segmentation based on multimodal medical image combinations allows radiomics features acquired from subregions to retain tumor heterogeneity, further improving the performance of radiomics. In this review, we provide a detailed summary of the current research on the radiomics of multimodal images of cancer and tumor subregion-based radiomics, and then raised some of the research problems and also provide a thorough discussion on these issues.

## Background

Cancer is a leading public health concern globally. Genetic biomarkers are critical for the suitable treatments for cancer patients 1. For example, IDH mutation 2-4, BRCA mutations 5,6, and EGFR amplification 7,8 have been widely reported for accurate diagnosis and precise treatment of cancers. However, the identification of biomarkers is based on the pathological tissues of cancers. Limited by the unique anatomy of some cancers, the method of obtaining tumor tissue usually involves risky surgery or stereotactic biopsy. Therefore, not all patients with cancer undergo genomic analyses. Even when genomic analysis is performed, the sampling is usually a one-time and single-site process. In addition, most cancer types are highly heterogeneous 9. The results of genomic analysis are highly susceptible to sampling errors and observer variability 10.

Radiomics is the process of converting medical images into mineable high-dimensional data 11,12. It has helped medical imaging expand from diagnostic aid to a clinical decision aid in the context of personalized medicine 13. Radiomics involves combining imaging data with patient characteristics, including clinical data 14, genomics 15,16, and drug response 17,18, to improve the functionality of decision models 19. Radiomics is generally considered a good solution to these issues: First, radiomics can help guide patients on the need for further genetic sequencing, as it can derive information from all parts of the tumor images 19; Second, the correlation between radiomics and genomics can guide biopsy site selection to address the high error rate of histopathology due to sampling errors 20,21; Third, radiomics can accurately predict valuable biomarkers in cancers, such as IDH mutations in glioma 22-24, and EGFR amplification in lung cancer 25.

**Figure 1:**
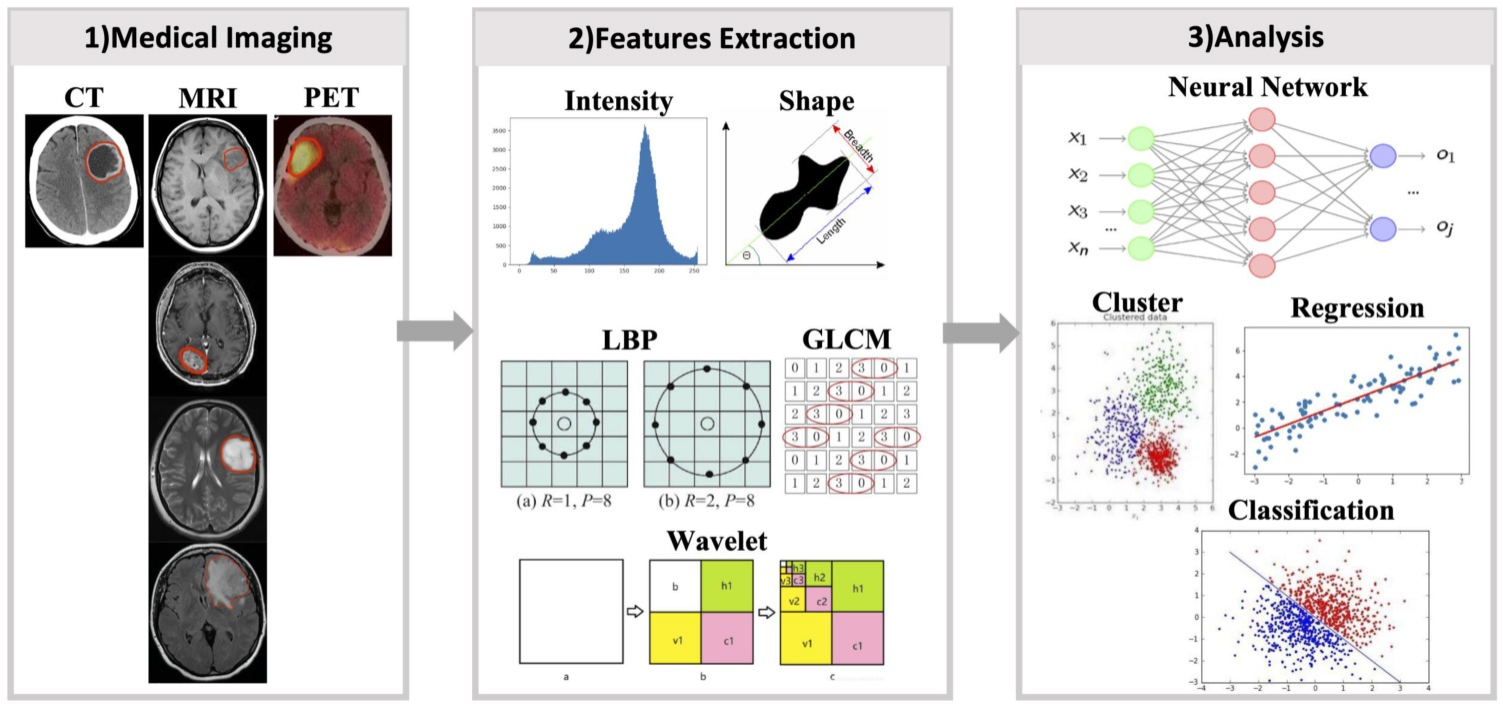
** Shortcomings of current radiomics research.** (Blue arrow) Different medical images can each obtain partial information about the tumor, and a large number of current radiomics studies tend to use only single modality images, and for MRI even only a few of its sequences are used, resulting in only a small fraction of tumor information being used. (Red arrow) Tumors are heterogeneous, but current radiomics tends to extract radiomics features from the entire tumor region on the image, thus making the information of tumor heterogeneity lost.

With the increasing popularity of radiomics, its application in cancer research is not only for genetic markers but also for patient survival, drug treatment effects, and other similar classification. However, some problems were also encountered. Previously, single-modality or single-sequence image was usually used for radiomics studies. But one modality or sequence can only reflect certain characteristics of the tumor. Therefore, the radiomics features extracted from single-modality images inevitable miss some tumor information, which will affect subsequent analysis. In addition, almost all cancers highly display intratumoral heterogeneity 9, and their medical images contain such heterogeneous information. However, many current radiomics studies have extracted radiomics features from the entire tumor region, which inevitably loses heterogeneous information in tumor images. These issues have been gradually considered. Multi-modality/sequence images combination have been used for radiomics studies and have yielded better results than single modality or sequence 26. Benefiting from the combination of multi-modality/sequence images, tumors have been segmented into regions reflecting different biological properties, and radiomics studies based on these regions have further improved the capabilities of radiomics 27. To make radiomics an effective diagnostic, prognostic, and predictive tool for cancer, it is important to understand advances in radiomics. This review describes recent advances in multimodality/multisequence studies in radiomics, the challenges that may be involved, and an outlook on the future of radiomics **(Fig. 1).**

## Progress of radiomics

**Figure 2:**
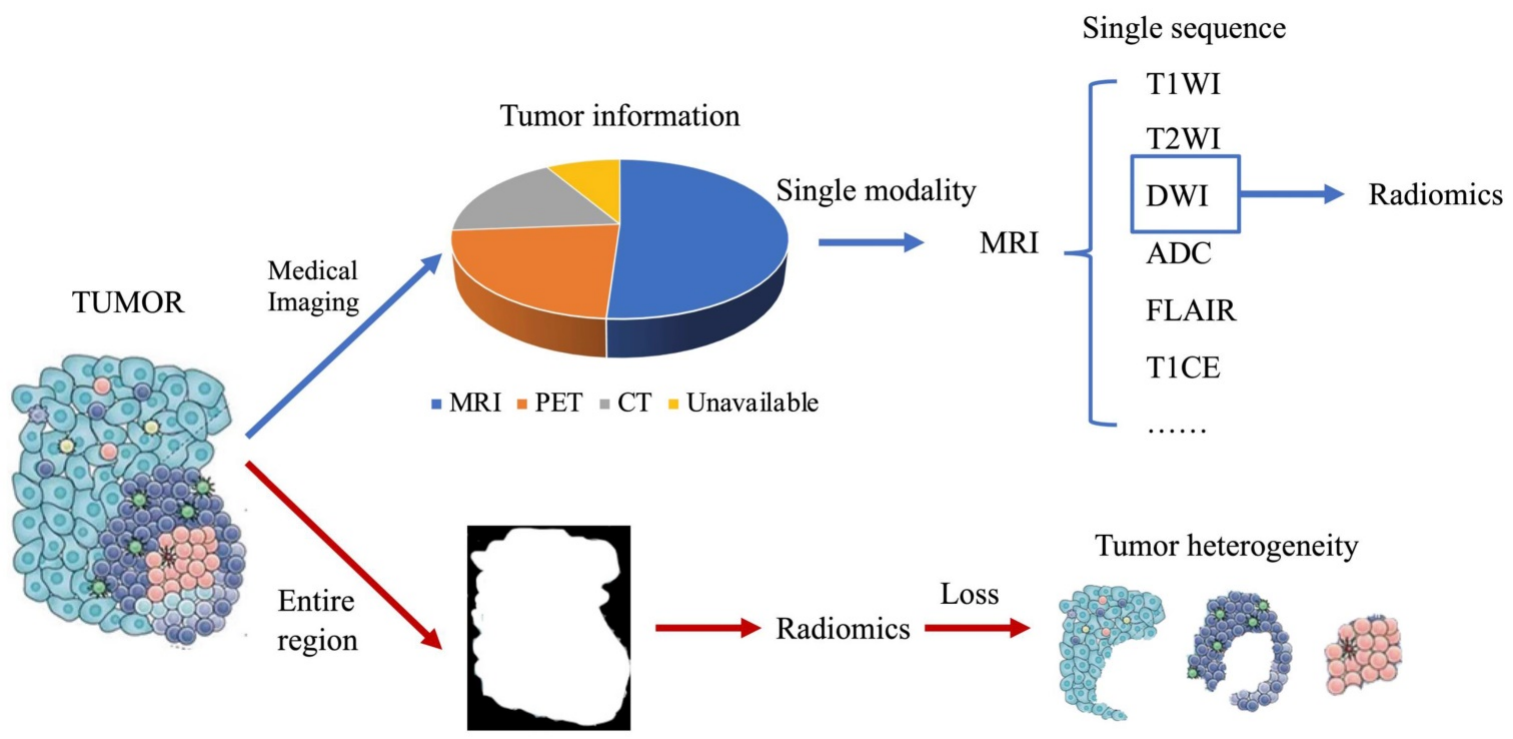
** Diagram of radiomics process.** 1) Radiomics first requires obtaining medical images of the patient and segmenting the target area. 2) Next step, various types of radiomics features were extracted from the images using a radiomics approach. 3) Using radiomics features, the models are constructed by machine learning or deep learning methods.

Radiomics is the process of extracting, analyzing, and building predictive models for radiomic features associated with predictive targets, and quantitative mapping between medical images and targets 28. It comprises a series of steps, including image acquisition, registration, segmentation, feature extraction, feature dimensionality reduction, and predictive modelling. 1) Image acquisition is the acquisition of clinical images from conventional imaging tools, such as computed tomography (CT), magnetic resonance imaging (MRI), and positron emission tomography (PET). 2) Registration involves establishing spatial correspondences between different image acquisitions 29. 3) Segmentation is the process of using clinical, imaging, and pathological knowledge to delineate the region of interest (ROI) in medical images. 4) Feature extraction is mining high-dimensional features from ROIs. 5) Feature dimensionality reduction involves cleaning redundant, irrelevant, or useless features. 6) Building robust predictive models is the ultimate purpose of radiomics research, which can help make clinical judgments and decisions **(Fig. 2)**.

Medical imaging commonly used in clinical includes X-ray, ultrasound, CT, MRI, and PET. Each type of imaging technique is suitable for various diseases. For example, for lung diseases, X-rays are usually selected first and CT is used to obtain more information; MRI are more common for brain and spinal cord diseases. Different MRI sequences can provide the most accurate data on different types of data for patients. Although there are radiomics studies of X-rays 30 and ultrasound 31, the main research of radiomics is focused on CT, MRI, and PET. Therefore, this study mainly focused on radiomics studies of these three imaging types **(Table 1)**.

### MRI

MRI can be classified into structural MRI, functional MRI, diffusion-weighted imaging (DWI), susceptibility-weighted imaging (SWI), and other medical and physiological applications. Different types of MRIs have different pathological and physiological characteristics. Therefore, MRI has received considerable attention in radiomics research, especially for precise diagnosis 32-34, treatment response prediction 35-37, molecular typing 38-40, and prognostic analysis 41,42.

Compared with models built by radiomics features only from structural MRI, it has been found that combining structural MRI with other MRI sequences can build better models. For example, in gliomas, radiomic features can be extracted from T1 contrast enhancement (T1CE), T2-weighting (T2WI), and apparent diffusion coefficient (ADC). The nomogram established by integrating the radiomics features extracted from these three MRIs had the best predictive value, which was higher than that established by any single sequence 26. Combining six types of MRI sequences (T2WI, DWI, ADC, fractional anisotropy, and mean kurtosis) for radiomics can accurately distinguish glioblastoma and low-grade glioma with an accuracy rate as high as 91% 43. A model for determining glioma grade was constructed with radiomics features from seven different sequences (T1-weighted (T1WI), T1CE, T2WI, FLAIR, diffusion tensor imaging (DTI), diffusion perfusion imaging, and 1H-MR spectra) achieving 95.5% accuracy, 95% sensitivity, 96% specificity, and 95.5% AUC 44. Pseudo-progression is a diagnostic challenge for glioblastoma after early treatment, and AUC of predictive model built by 12 radiomics features from T1CE, FLAIR, ADC, and cerebral blood volume (CBV) mapping is 0.90, which is much higher than those built using only features from conventional sequences (AUC=0.76), ADC (AUC=0.78), and CBV (AUC=0.8) 45. It is widely known that IDH mutations play a pivotal role in gliomas. The AUC of the model for predicting IDH mutation status using conventional MRI radiomics features was 0.835, which increased to 0.9 after combining the radiomics features of DTI 46. In another radiomics study to predict IDH mutations, the model achieved an accuracy of 0.823 by using radiomics features from T1CE, T2WI, and ASL images 47. The AUC of the nomogram to predict IDH mutations using T1CE, FLAIR, and ADC radiomics features combined with age was 0.913 48. In addition, other key signaling pathways can also be assessed using MRI radiomics. Using radiomics features of conventional MRI and DWI, the AUC of the model predicting RTK in glioma was 0.88, TP53 was 0.76, and RB1 was 0.81, which was greater than that of the model using only conventional MRI 49.

In addition to gliomas, the combination approach has been widely used for other tumors. The radiomics features from T2WI, T1WI, diffusion kurtosis imaging (DKI), ADC, and dynamic contrast-enhanced (DCE) pharmacokinetic parameter maps were used to build breast cancer diagnosis models, and the best model (combination of T2WI, DKI, and DCE) AUC was 0.921, accuracy was 0.833, and the AUCs of the model based on single T1WI, T2WI, ADC, DKI, and DCE pharmacokinetic parameter maps were 0.730, 0.791, 0.770, 0.788, and 0.836, respectively 50. To predict whether breast cancer will produce a pathologic complete response to neoadjuvant chemotherapy, a prediction model jointly built using the radiomics features of T2WI, DWI, and T1CE had an AUC of 0.79 in the training set and reached 0.86 in the validation set 51. In prostate cancer, the need for biopsy at PSA levels of 4-10 ng/mL is a clinical question, and a prediction model built using radiomic features extracted from T2WI, DWI, and T1CE achieved an AUC of 0.956 in the training set and 0.933 in the validation set 52. Good performance was obtained for models predicting prostate cancer with a Gleason score of 8 or higher (AUC = 0.72), built from radiomics features extracted from T2WI, ADC, and DKI; better performance was also obtained for models predicting prostate cancer with a Decipher score of 0.6 or higher (AUC 0.84) 53.

**Figure 3:**
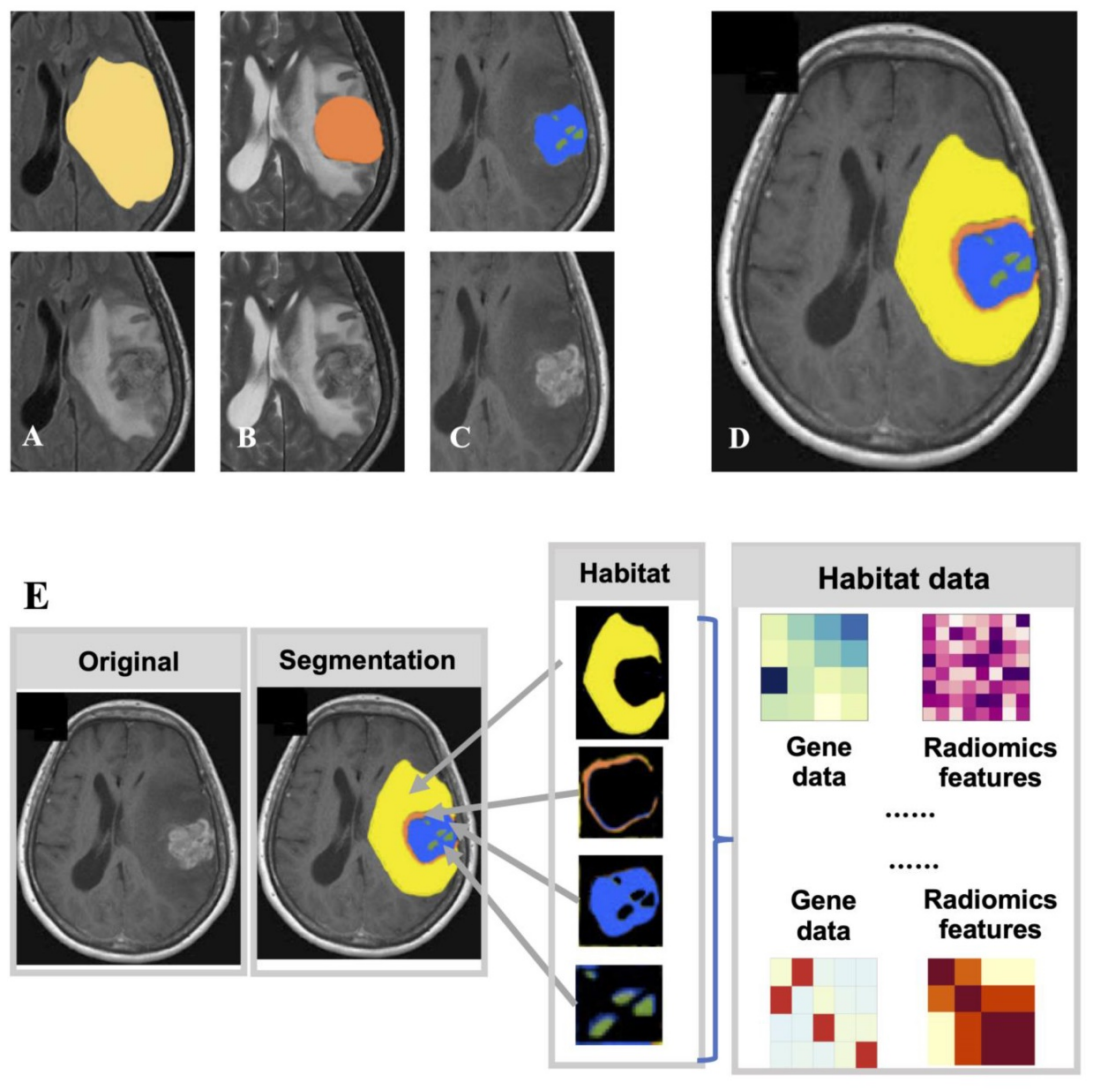
** Radiomics research based on subregion of tumor generated by multimodal medical image.** (A) is FLAIR, from which the edematous part (yellow), (B) is T2, from which the overall tumor core can be seen, and (C) is T1CE, from which the enhancing part (blue) and necrotic part (green) of the tumor core can be obtained. (D) After superimposing the labels together, the final habitat segmentation results can be obtained, where the non-enhanced part of the tumor core (red) is the overall tumor core minus the enhanced and necrotic parts. (E) After segmenting the tumor area into different subregions, radiomic studies were performed from each subregion.

Because of the unique biophysical principles that control signal generation, MRI sequences provide a non-invasive way of physiologically reflecting different local microenvironments. Solid tumors are genetically and physiologically heterogeneous. Different physiological regions exert different selective pressures, resulting in the growth of tumor cell clones with an adapted genome/proteome. These regions are also called "Habitats" 27, and research on radiomics has gradually shifted from the entire tumor area to the habitats **(Fig. 3)**. T1WI, FLAIR, and ADC can divide the ROI into two habitats, the tumor and the edema around the tumor, and then extract radiomics features from the two habitats. The AUC of the glioma MGMT methylation prediction model constructed using the combination of radiomics features from the two habitats reached 0.925 in the training set and 0.902 in the validation set 54. To predict the main pathological growth pattern of liver metastases from colorectal cancer, radiomics features were extracted from multiple MRI sequences (T1WI, T2WI, DWI, ADC, and T1CE) from the tumor-liver interface region, i.e., the region between the tumor margin expanding outward by 2 mm and shrinking inward by 2 mm, and the prediction model incorporating these radiomics features had an AUC of 0.912. superior to the model constructed from radiomics features extracted from tumor regions (AUC=0.879) 55. A total of nine MRI sequences of sagittal T2WI, axial T1WI, axial T2-FS, DWI (b=0 and b=800), ADC, and T1CE (sagittal, axial, and coronal) in advanced cervical cancer were manually outlined by the radiologists, the radiomics features were extracted, and the AUC of the model constructed by combining the radiomics features of multiple sequences reached 0.82 56. To assess the survival risk of glioblastoma and predict its progression-free survival, T1CE, T1WI, and FLAIR sequences were used to delineate and annotate the necrotic core, enhancement part, and surrounding edema of glioblastoma. Radiomics features were extracted from these features, which can be used to accurately stratify the survival risk of patients with glioblastoma 57.

### PET

Cancer cells usually receive more imaging agents, resulting in malignant tumors appearing as bright spots on PET. The amount of imaging agent taken by a malignant tumor depends on the metabolic level of the malignant tumor; therefore, PET radiomics is also called metabolic radiomics.

PET is usually combined with CT, the most common clinical practice is PET-CT. Using the radiomics features from PET and CT in patients with suspected lung cancer, the AUC of the established predictive nomogram was 0.96, which is far higher than the 0.79 of only CT radiomics features, and 0.93 of only PET radiomics features 58. For accurate differentiation between active pulmonary tuberculosis and lung cancer, three radiomics signatures were constructed. The PET radiomics signature yielded AUC of 0.79 (95 % CI: 0.67-0.86]; the CT radiomics signature displayed higher discrimination performance relative to the PET radiomics signature (AUC, 0.86; 95 % CI, 0.79-0.91); the PET/CT radiomics signature incorporating both PET and CT radiomics features further improved the discriminatory ability with the highest AUC (0.91, 95 % CI, 0.84-0.95) 59. To distinguish squamous cell carcinoma from adenocarcinoma of lung, the combined model, comprising 7 PET radiomics and 3 CT radiomic parameters, had the highest predictive efficiency and clinical utility in predicting the non-small cell lung cancer subtypes compared to the use of these parameters alone in both the training and validation sets (AUCs (95% CIs) = 0.932 (0.900-0.964), 0.901 (0.840-0.957), respectively) (p < 0.05) 60. For prediction of the prognosis of patients with NPC, combining PET and CT features with clinical parameters showed equal or higher prognostic performance than models with PET, CT, or clinical parameters alone (C-index 0.71-0.76 vs. 0.67-0.73 and 0.62-0.75 vs. 0.54-0.75 for training and validation cohorts, respectively), while the prognostic performance was significantly improved in the locally advanced regional cohort (C-index 0.67-0.84 vs. 0.64-0.77, p value 0.001-0.059) 61. To predict HPV status in oropharyngeal squamous cell carcinoma, radiomic features were extracted from primary tumor lesions and metastatic cervical lymph nodes on PET and non-contrast CT scans. Single PET or CT models yielded similar classification performance without significant difference in independent validation; however, models combining PET and CT features outperformed single-modality PET- or CT-based models, with AUC of 0.78, and 0.77, respectively, in cross-validation and independent validation, respectively 62.

Compared to PET/CT combinations, PET/MRI combinations emerged much later and are currently less used; however, some progress has been made in dual-modality radiomics studies of PET and MRI. To investigate the potential of combined textural feature of contrast-enhanced MRI (CE-MRI) and static O-(2-[18F]fluoroethyl)-L-tyrosine (FET) PET for differentiating between local recurrent brain metastasis and radiation injury, radiomic features were extracted from above two imagines individually. CE-MRI texture features had a diagnostic accuracy of 81% (sensitivity, 67%; specificity, 90%). FET PET textural features revealed a slightly higher diagnostic accuracy of 83% (sensitivity, 88%; specificity, 75%). However, the highest diagnostic accuracy was obtained CE-MRI and FET PET features were combined (accuracy, 89%; sensitivity, 85%; specificity, 96%) 63. Approximately 15-30% of locally advanced rectal cancers (LARC) will produce a pathologic complete response (pR) to neoadjuvant therapy. To predict whether LARC responds to neoadjuvant therapy, radiomic features were extracted from PET and MRI. A model containing 6 radiomics features (5 from PET and 1 from MRI) yielded the highest predictability in distinguishing between pR+ and pR- patients (AUC = 0.86; sensitivity = 0.86; sensitivity = 86%, and specificity = 83%) 64. For prediction of hormone receptor status and proliferation rate in breast cancer, a model constructed by combining radiomics features of PET and MRI achieved the best results for AUC (estrogen receptor, 0.87; progesterone receptor, 0.88; Ki-67, 0.997) 65.

As mentioned above, tumors are highly heterogeneous, and this heterogeneity can be reflected in PET images. Their general assessments are made using the intratumoral heterogeneity index (SUVmax/SUVmean) which can help predict more accurately the prognosis of patients with tumors 66, 67. In addition, similar to MRI, PET can be used to segment tumors into subregions (“Habitats”) with different physiological characteristics based on imaging principles. The radiomics features from the subregions retained the heterogeneous information of the region and avoided the loss of this heterogeneous information. To predict PFS in patients with NPC, each tumor in the PET/CT imaging was partitioned into several phenotypically consistent subregions. For each subregion, 202 radiomic features were extracted to construct the models. Three subregions (denoted as S1, S2, and S3) with distinct PET/CT imaging characteristics were identified. The prognostic performance of the model from S3 outperformed the model from the whole tumor (C-index, 0.69 vs. 0.58; log-rank test, p < 0.001 vs. p = 0.552) 68. To preoperatively discriminate between non-small cell lung cancer and benign inflammatory diseases, PET/CT was separated into variant subregions based on the adapted clustering method. The AUC of the subregion-based PET/CT radiomics models was 0.7270 ± 0.0147, which showed a significantly improved discrimination performance compared to conventional methods (p <.001) 69. Radiomic features were extracted from whole PET, CT, and subregions to estimate the prognosis of patients with locally advanced cervical cancer treated with chemoradiotherapy. The radiomics signatures that included habitat features achieved significantly higher C-indexes of 0.78 and 0.76 for PFS estimation, and 0.83 and 0.78 for OS estimation in the training and test cohorts, respectively, compared with radiomics signatures without habitat features with C-indexes of 0.72 and 0.68 for PFS estimation (P = .004, z test). and 0.79 and 0.72 for OS estimation (P = .048, z test) 70.

### CT

CT reflects the spatial distribution of tissue strength in the detected area. It is the most commonly used medical radiological examination in clinical practice. Radiomics of CT in cancer has been extensively researched 71-73.

Recently, radiomics studies combining plain CT with contrast-enhanced CT (CE-CT) have achieved better results than plain CT alone. When using CE-CT and CT plain scans to predict EGFR mutation status in patients with non-small cell lung cancer, the general radiomics signature (CT+CE-CT) yielded the highest AUC of 0.756 and 0.739 in the two test sets, and the performance of the general radiomics signature was always similar to or higher than that of models built using only CT or CE-CT features 74. CE-CT can be divided into different phases, which contain different tumor information. To develop and validate a radiomics-based nomogram for preoperatively predicting grade 1 and grade 2/3 tumors in patients with pancreatic NETs, radiomic features were extracted from arterial phase (AP) and portal venous phase (PVP) CT images. The fusion radiomic signature (CT+CE-CT) achieved optimal performance in both the training (AUC 0.970; 95% CI 0.943-0.997) and validation (AUC 0.881; 95% CI 0.760-1) cohorts 75. To predict histopathologic growth patterns (HGPs) in colorectal liver metastases (CRLMs) using a radiomics model, radiomic features were extracted from the AP and PVP CT images. The phase-fused radiomics signature demonstrated the best predictive performance for distinguishing between replacement and desmoplastic HGPs (AUCs of 0.926 and 0.939 in the training and external validation cohorts, respectively) 76. A total of 384 radiomics features were extracted from AP or PVP images to preoperatively discriminate pancreatic ductal adenocarcinoma (PDAC) in stages I-II and III-IV, and predict overall survival. The AP+PVP radiomics signature showed the best performance among the three radiomics signatures (training cohort: AUC = 0.919; validation cohort: AUC = 0.831) 77.

In clinical practice, CT and MRI are the most commonly used imaging modalities. The performance of combined CT and MRI radiomics is usually better than that of either alone. To predict the treatment response to neoadjuvant chemotherapy for LARC, radiomics features were extracted from CT, ADC, T1CE, and high-resolution T2-weighted imaging (HR-T2WI). For an individual sequence, the HR-T2WI model performed better (AUC = 0.859, ACC = 0.896) than CT (AUC = 0.766, ACC = 0.792), T1CE (AUC = 0.812, ACC = 0.854), and ADC (AUC = 0.828, ACC = 0.833) models in the validation set; the combined radiomics model (AUC = 0.908, ACC = 0.812) had a better performance than the individual T1CE, HR-T2WI, and ADC models 78. To build a predictive model of lymphatic vascular infiltration (LVI) in rectal cancer, radiomic features were extracted from T2WI, DWI, and CE-CT. The combined model achieved the highest AUC of all single-modal models (AUC = 0.884 and 0.876, respectively), with high sensitivity and specificity in the training and validation cohorts (sensitivity = 0.938 and 0.929, specificity = 0.727 and 0.800, respectively) 79. To predict pCR in patients with rectal cancer after neoadjuvant treatment, a radiomics model based on pretreatment CT was built first. Even the CT radiomics model yielded the highest AUCs of 0.997 [95% CI 0.990-1.000] in the primary cohort and 0.822 [95% CI, 0.649-0.995] in the validation cohort. When the MRI-based radiomics signature was added to the previous CT radiomics model, the performance of the integrated model (CT-MRI) was significantly better than that of CT (P = 0.005) or MRI (P = 0.003) alone (AIC: 75.49 vs. 81.34 vs. 82.39%) 80.

CT is often combined with PET for radiomic studies of tumor subregions, as summarized in the PET section above. Theoretically, CT and CE-CT, or CT and MRI could also be combined with each other for tumor subregion studies, but to the best of our knowledge, we have not found any literature on this yet.

## Discussion

**Figure 4:**
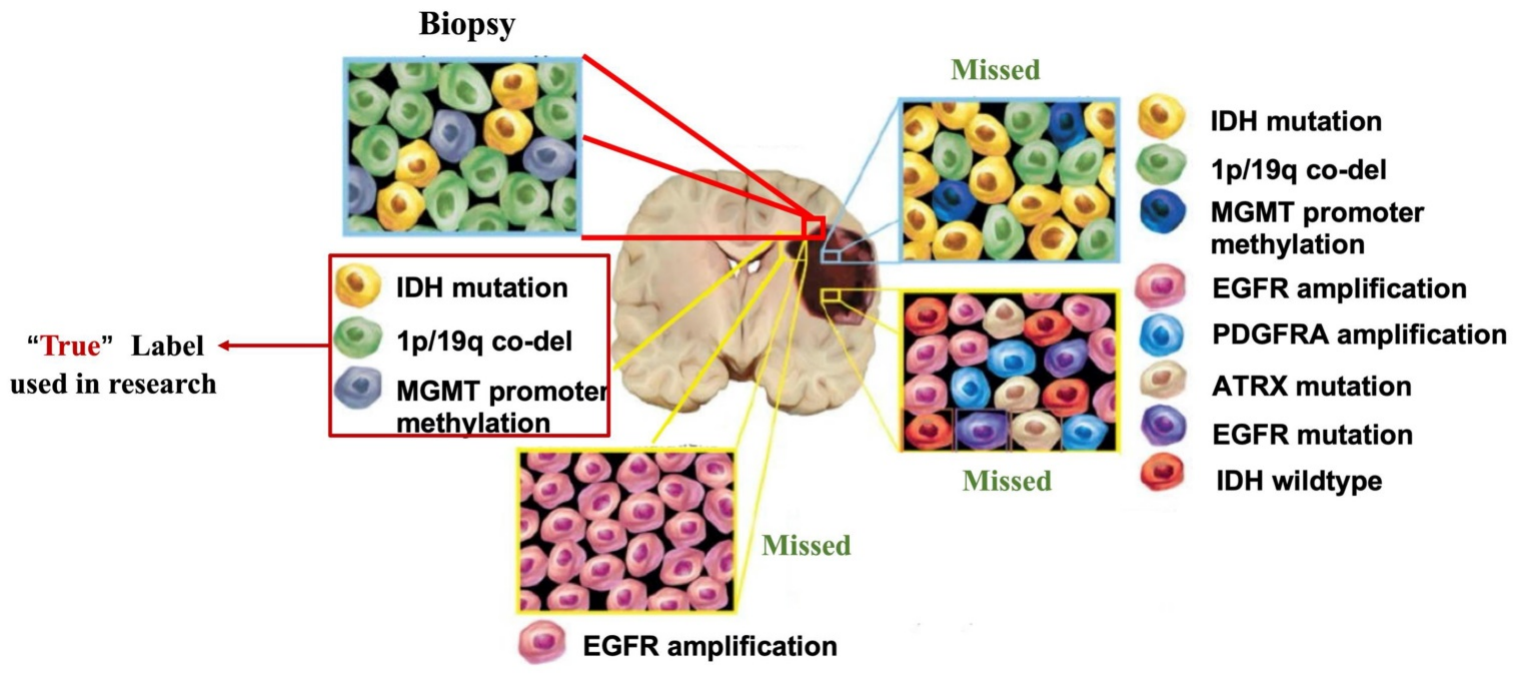
** Tumor heterogeneity can lead to bias in genomics data.** Tumors are highly heterogeneous, with cells of different genotypes in different subregions. “Biopsy”: one site can only obtain the genotypes of cells in a localized region of the tumor. “Missed”: The genotypes of other parts of the tumor are lost. However, most of the current studies have used this biased data as a "true" label.

By combining multimodal images, radiomics can obtain more comprehensive image information and better radiomic models, and even segment "subregions" with different biological characteristics and perform more refined radiomic analysis based on these subregions. However, it must be noted that the current tumor genomics data, both in public databases and clinical practice, are derived from single-site sampling of tumors **(Fig. 4)**. The genomic data obtained inherently ignore the heterogeneity of tumors and have the potential for substantial bias 81. The true efficacy of radiomics models trained using biased data is questionable. For example, in a radiomics study to predict the presence of IDH mutations in gliomas, if the labels in a supervised learning model are incorrect because of the genetic heterogeneity of the gliomas, then the model is bound to be unreliable. Tumor heterogeneity will also indirectly affect the study of a range of factors, such as treatment response, prognosis, and tumor recurrence. An ideal way to solve this problem is to obtain heterogeneous genomic data of the tumor. However, it is unrealistic based on technology and costs. Therefore, a method to obtain the genomic heterogeneity of tumors as realistically as possible is an unavoidable challenge in the continued development of future radiomics. Medical images can analyze different subregions to inform tissue biopsy sites 82. The more accurate conclusions can be drawn by analyzing genomic information sampled from different tumor subregions. The reliability of different subregions in radiomics can be further verified using genomic data obtained from different subregions. However, to date, there is no public database with both genomic and imaging data of tumor subregions.

**Figure 5:**
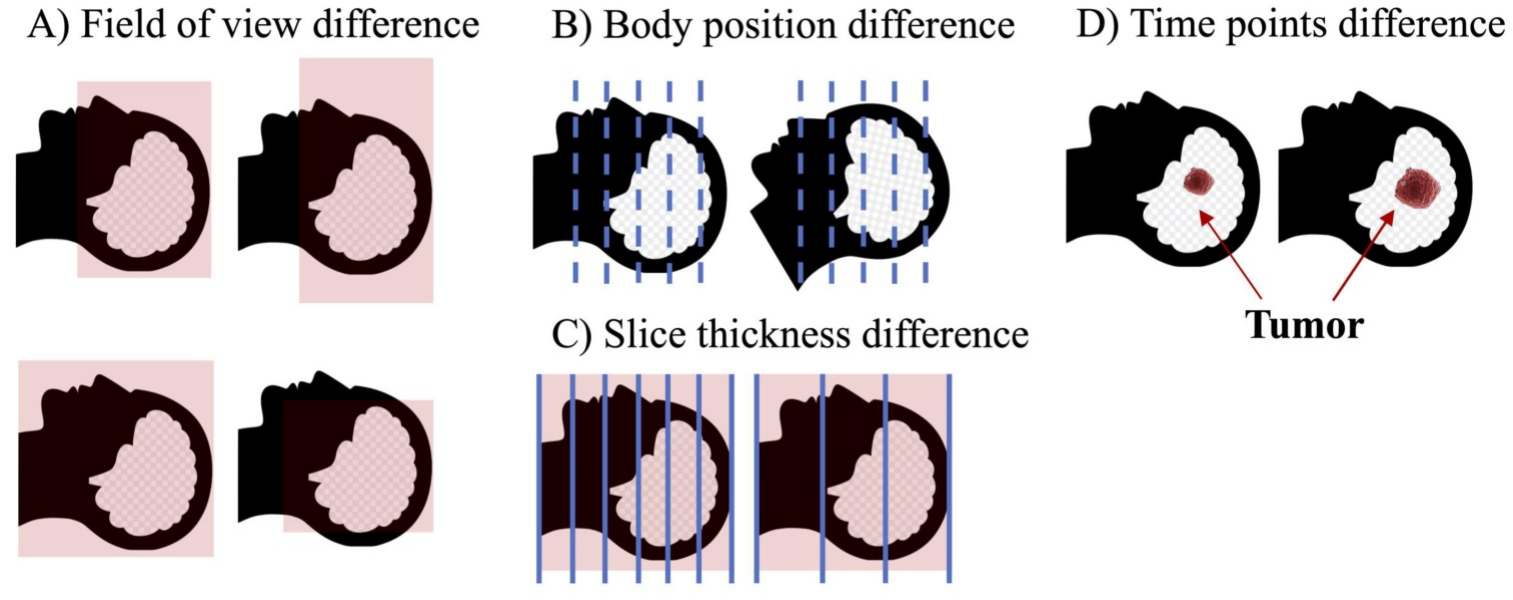
** Factors leading to differences in the spatial structure of medical images of different modalities.** (A) Field of view may be different for different modality images at the time of examination. (B) Images of different modalities are often not completed simultaneously, and the patient's position cannot be guaranteed to be identical during multiple imaging examination. (C) Images of different modalities usually have different slice thicknesses. (D) The same patient may be imaged at different times, which may result in structural changes for some rapidly progressing tumors.

Although the combination of multimodal images has certainly helped the advancement of radiomics, the implementation process still has many problems. For example, for studies that require the combination of CT and MRI, it is likely that medical images of different modalities will have different spatial structure because they usually come from different points in time, and the patient's position, field of view of the device, and number of slices scanned may differ, even if the patient is the same. In this case, it is necessary to align medical images of different modalities **(Fig 5)**. However, although many image alignment tools have been developed, they do not always yield ideal alignment results 83,84, especially for diseases such as tumors, which can cause significant changes in normal anatomy. Cancer-specific tumor alignment tools have been used to address this problem 85, but there are so many different cancer types that the cost of this approach remains exorbitant. Development of a universal and reliable registration tool is a possible solution to this problem.

In addition, multimodal images also indicate that the researcher must outline the ROIs for multiple images. For researchers who manually outline ROIs, this workload has undoubtedly increased by several times. Even for researchers who use or develop their own semi-automatic or fully automatic segmentation tools, more effort is needed to develop different segmentation tools, as a single segmentation tool is unlikely to cope with images of different modalities. Radiomics studies of multimodal images require more reliable segmentation. The current segmentation for multimodal images involves multiple iterations of the segmentation of a single-modal image 86. Therefore, each error in multimodal image segmentation magnifies the error in the final segmentation result 87. Such errors have a greater impact on “subregion” based imaging studies, where even small segmentation errors may determine the presence or absence of certain “subregions.” Addressing this issue should be a future focus in radiomics.

To verify the capabilities of radiomics and expand its application range, “big” data with a large number of various data types are required 88. Powerful public databases such as TCIA 89 and TCGA 90 are effective solutions for this. However, the implementation of big data is difficult. The data in public databases come from different institutions, such as TCIA, resulting in variable quality of their data information. While big data can draw reliable conclusions from relatively "dirty" data, in the case of radiomics, the different parameters of the image acquisition or the noise in the image can cause serious interference with the radiomics features 91,92, which will inevitably affect the model's ability for generalization of other databases 93. To solve this problem, it is necessary to develop worldwide database access standards. In addition, it is necessary to utilize this standard to clean the data in existing public databases. However, despite the existence of public databases, the amount of data in these databases is extremely limited. Although many institutions have a large amount of image data, they are still independent of each other. Making use of all data from all sources can greatly expand the number of samples and produce more reliable results. This can be accomplished by facilitating the federation of medical centers, allowing them to request access to each other's data, or by commissioning a trusted third-party organization that can manage the data of all participating institutions. Although it is costly and difficult to unite institutions or find a third party to build a high-quality public database, the benefits are enormous.

The relationship between radiomics and clinical symptoms has been widely documented, and correlations between radiomics and other data types, such as genomics, transcriptomics, proteomics, and metabolomics, are major directions for future development. The correlation between radiomics and genomics has been extensively studied in cancers. Radiomics features can also be used to represent transcriptomics. Research on the combination of radiomics with proteomics and metabolomics is just beginning, and more effort is required. The combined research of radiomics and another kind of omics, the combined research of multiple omics, is an area worthy of future research. However, omics data are independent of each other, that is, samples with genomic data may not have radiomics data, or samples may have radiomics data but lack transcriptomic data. A high-quality multi-omics database is a good way to break this isolation, but the cost is huge.

## Conclusions

In this review, we summarized the current advances and hotspots in radiomics research. Multimodality/multisequence medical image fusion radiomics and habitat-based radiomics research fully consider the uniqueness of each medical imaging modality, compensate for the shortcomings of omitted information in previous radiomics studies, and greatly improve the prediction accuracy of radiomics, which is a future development trend. With the improvement in radiomics technology, expansion of public databases, and advancement of deep learning algorithms, radiomics will definitely play an important role in future clinical diagnosis, treatment, and prognosis.

## Figures and Tables

**Table 1:** Information of radiomic studies in human cancer

Years	Cited reference	Tumor Types	Imaging combination
2019-2020	26, 43-53	Glioma, Breast cancer, Prostate cancer	Multimodal MRI sequences
2019-2021	58-62	Lung cancer, Nasopharyngeal carcinoma, Oropharyngeal squamous cell carcinoma	PET and CT
2018-2021	63-65	Brain metastasis, Rectal cancers, Breast cancer	PET and MRI
2020-2021	78-80	Rectal cancers	MRI and CT
2019-2021	74-77	Lung cancer, Pancreatic cancer, Colorectal liver metastases	CT and CE-CT
2015-2021	54-57, 66-70	Glioma, Colorectal cancer, Cervical cancer, Nasopharyngeal carcinoma, Lung cancer	Subregion

## Abbreviations

CT: computed tomography
MRI: magnetic resonance imaging
PET: positron emission tomography
ROI: region of interest
DWI: diffusion-weighted imaging
SWI: susceptibility-weighted imaging
T1CE: T1 contrast enhancement
T2WI: T2-weighting
ADC: apparent diffusion coefficient
CBV: cerebral blood volume
DKI: diffusion kurtosis imaging
DCE: dynamic contrast-enhanced
CE-MRI: contrast-enhanced MRI
FET PET: O-(2-[18F]fluoroethyl)-L-tyrosine (FET) PET
LARC: locally advanced rectal cancers
HGPs: histopathologic growth patterns
CRLMs: colorectal liver metastases
AP: arterial phase
PVP: portal venous phase
PDAC: pancreatic ductal adenocarcinoma
HR-T2WI: high-resolution T2-weighted imaging
RTK: receptor tyrosine kinase
